# Supplementary material for: Identification of photoperiod-induced specific miRNAs in the adrenal glands of Sunite sheep (Ovis aries)
Source: Front Vet Sci. 2022 Jul 22;9:888207. doi: 10.3389/fvets.2022.888207 (PMC9354845; doi:10.3389/fvets.2022.888207)
Supplement: Supplementary file 1 [file Data_Sheet_1.ZIP › Supplementary file/miRDeep2 file.zip!/miRDeep2 file/Novel_Structure/LP42A1/Novel_284.pdf]

Provisional ID	:	NC_019470.2_21889
Score total	:	18.9
Score for star read(s)	:	3.9
Score for read counts	:	11.9
Score for mfe	:	1.5
Score for randfold	:	1.6
Score for cons. seed	:	
Total read count	:	35
Mature read count	:	34
Loop read count	:	0
Star read count	:	1

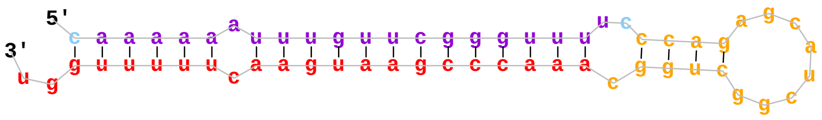

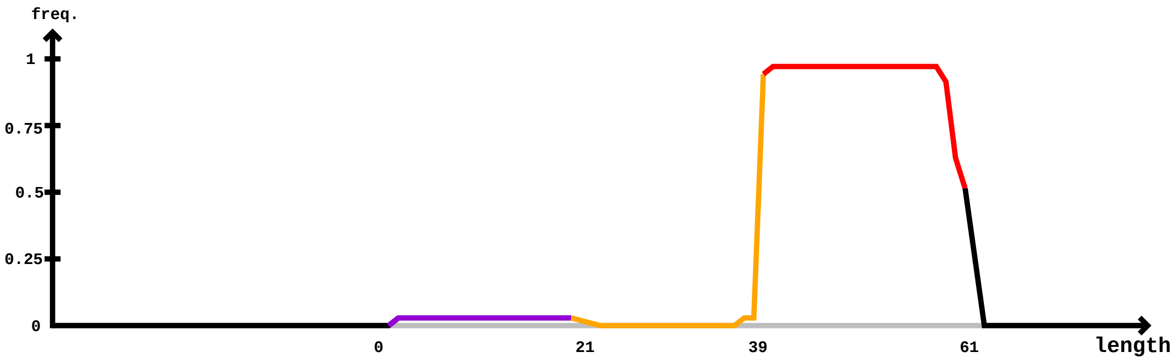

Star Mature

```
5'- guguugaucuaacacacuauaucucagguuggucaaaaaauuuguucggguuuucccagagcaucggcuggcaaacccgaaugaacuuuuuggucaacucacuacucuggca  -3'  obs
    guguugaucuaacacacuauaucucagguugguucaaaaaauuuguucggguuuucccagagcaucggcuggcaaacccgaaugaacuuuuuggucaacucacuacucuggca       exp
    ((((((...))))))...........(((((((((((((.(((((((((((((..((((........)))).))))))))))))).))))))))))))).............   reads   mm   sample
    ..................................aaaaaGuuuguucggguuuu..........................................................   1       1    seq
    ......................................................................gcaaacccgaaugaacuuuuug....................   1       0    seq
    ........................................................................aaacccgaaugaacuuuuC.....................   1       1    seq
    ........................................................................aaacccgaaugaacuuuuuC....................   1       1    seq
    ........................................................................aaacccgaaugaacuuuuuU....................   2       1    seq
    ........................................................................aaacccgaaugaacuuuuuA....................   6       1    seq
    ........................................................................aaacccgaaugaacuuuuuUg...................   4       1    seq
    ........................................................................aaacccAaaugaacuuuuuggu..................   1       1    seq
    ........................................................................aaacccgaaugaacuuuuuggC..................   7       1    seq
    ........................................................................aaacccgaaugaacuuuuuggu..................   10      0    seq
    .........................................................................aacccgaaugaacuuuuu.....................   1       0    seq
```
